# Gliome database: a comprehensive web-based tool to access and analyze glia secretome data

**DOI:** 10.1093/database/baaa057

**Published:** 2020-07-31

**Authors:** Jong-Heon Kim, Su-Hyeong Park, Jin Han, Pan-Woo Ko, Dongseop Kwon, Kyoungho Suk

**Affiliations:** 1 Brain Science and Engineering Institute, Kyungpook National University, 680 Gukchaebosang-ro, Jung-gu, Daegu, 41944, Republic of Korea; 2 Department of Pharmacology, School of Medicine, Kyungpook National University, 680 Gukchaebosang-ro, Jung-gu, Daegu, 41944, Republic of Korea; 3 D&P BIOTECH, 807 Hoguk-ro, Buk-gu, Daegu, 41404, Republic of Korea; 4 Department of Neurology, Kyungpook National University Chilgok Hospital, 807 Hoguk-ro, Buk-gu, Daegu, 41404, Republic of Korea; 5 School of Software Convergence, Myongji University, 34 Geobukgol-ro, Seodaemun-gu, Seoul, 03674, Republic of Korea

## Abstract

Glial cells are phenotypically heterogeneous non-neuronal components of the central and peripheral nervous systems. These cells are endowed with diverse functions and molecular machineries to detect and regulate neuronal or their own activities by various secreted mediators, such as proteinaceous factors. In particular, glia-secreted proteins form a basis of a complex network of glia–neuron or glia–glia interactions in health and diseases. In recent years, the analysis and profiling of glial secretomes have raised new expectations for the diagnosis and treatment of neurological disorders due to the vital role of glia in numerous physiological or pathological processes of the nervous system. However, there is no online database of glia-secreted proteins available to facilitate glial research. Here, we developed a user-friendly ‘Gliome’ database (available at www.gliome.org), a web-based tool to access and analyze glia-secreted proteins. The database provides a vast collection of information on 3293 proteins that are released from glia of multiple species and have been reported to have differential functions under diverse experimental conditions. It contains a web-based interface with the following four key features regarding glia-secreted proteins: (i) fundamental information, such as signal peptide, SecretomeP value, functions and Gene Ontology category; (ii) differential expression patterns under distinct experimental conditions; (iii) disease association; and (iv) interacting proteins. In conclusion, the Gliome database is a comprehensive web-based tool to access and analyze glia-secretome data obtained from diverse experimental settings, whereby it may facilitate the integration of bioinformatics into glial research.

## Introduction

Glial cells are the most abundant non-neuronal cells in the nervous system. They are a heterogeneous class of cells, comprising astrocytes, microglia, oligodendrocytes and ependymal cells in the central nervous system and Schwann and satellite glial cells in the peripheral nervous system. Glial cells mainly function in the homeostasis and surveillance of neurons and also support and nurture them (1–5). Accumulated evidence has shown that they actively regulate neuronal activity and morphology (6). Glia also participate in synapse remodeling, blood–brain barrier, neurogenesis and neuroinflammation (7, 8).

Glial cells communicate with each other or neurons through the release of signaling molecules, predominantly secretory proteins. Glial secretory proteins play essential roles in mediating various biological processes under healthy or diseased conditions. For example, these proteins initiate intracellular signaling cascades leading to changes in gene expression and inducing cellular processes that include proliferation, differentiation, migration and protein secretion (1, 9).

Secretome is a term that denotes all the factors secreted by cells, such as soluble proteins, cytokines (10), growth factors (11), extracellular matrix proteins (12) and shed membrane receptors (13). The secretory proteins typically include a hydrophobic signal peptide at the *N*-terminus undergo their journey along the biosynthetic-secretory pathway through the endoplasmic reticulum and Golgi apparatus. These are eventually secreted into the extracellular space via the classical, non-classical, extracellular membrane vesicular (14) or exosomal pathways (15). Secretomics is a sub-field of proteomics that involves the analysis of the secretome including the liquid chromatography–mass spectrometry (LC-MS/MS) analysis, enzyme-linked immunosorbent assay (ELISA) or protein array (16, 17). Since secretome plays a myriad of roles ranging from homeostasis to biological regulation, such as intercellular cross-talk (12), coordination of biological activity (18) and disease development (19), secretomics has been a good source for characterizing and quantifying proteins secreted by a given cell under specific experimental conditions and a powerful strategy for discovering disease biomarkers (20–22).

**Figure 1:**
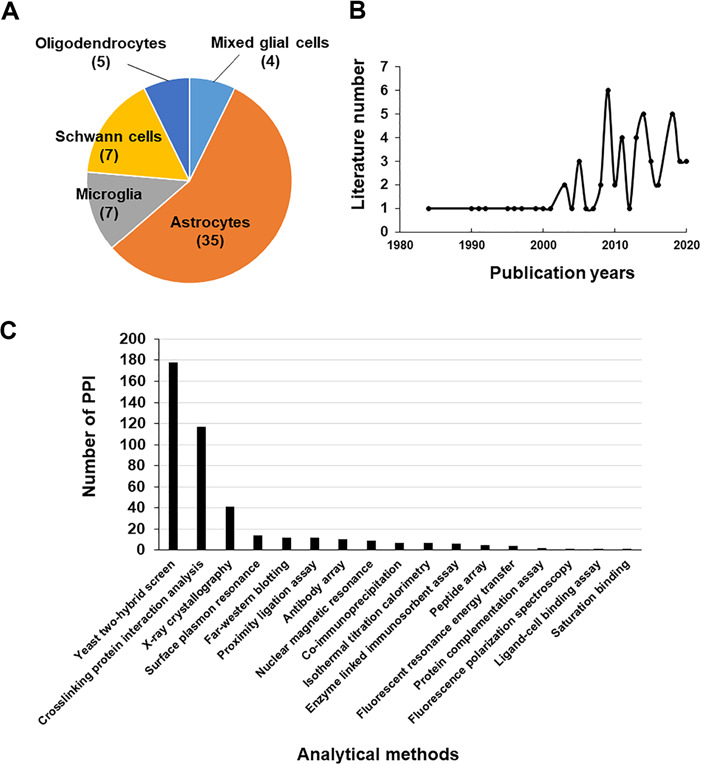
Literature used in the Gliome database. (**A**, **B**) The distribution of the literature according to glial cell type (A) or the publication year (B). (**C**) The number of PPIs classified by analytical methods.

**Figure 2:**
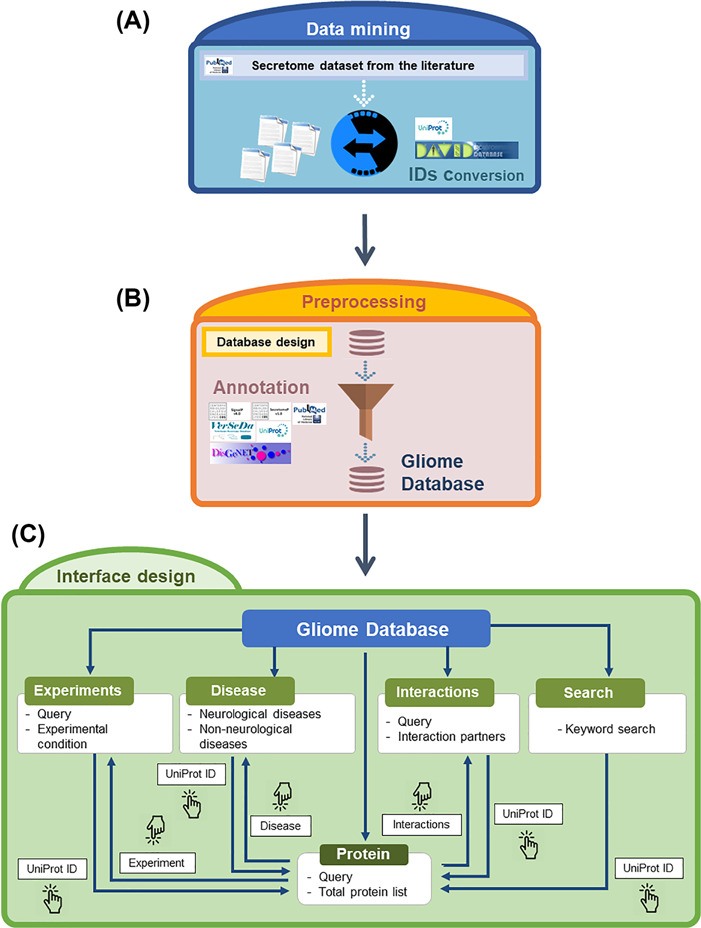
Overview of the Gliome database. (**A**) Data mining step to obtain the glia secretome information. The obtained protein names are converted into UniProt ID. (**B**) Information is entered according to the database format so that users can browse various types of information. At this step, various external databases were used alongside ours. (**C**) A diagram of navigation at the Gliome database. The protein identifiers of any types were converted into official gene name/UniProt ID.

**Table 1:** The websites of the public databases used in this work

Database	URL	Description
Google Scholar	https://scholar.google.com/	Google Scholar provides a simple way to broadly search for scholarly literature.
Scopus	https://www.scopus.com	Scopus is an extensive, multidisciplinary database of peer-reviewed literature.
PubMed	https://www.ncbi.nlm.nih.gov/pubmed/	PubMed is a free search engine to access primarily the MEDLINE database of references and abstracts on life sciences and biomedical topics.
SignalP	http://www.cbs.dtu.dk/services/SignalP/	The SignalP 5.0 server predicts the presence of signal peptides and the location of their cleavage sites in proteins.
SecretomeP	http://www.cbs.dtu.dk/services/SecretomeP/	The SecretomeP 2.0 server performs *ab initio* prediction of classical vs. non-classical secretion.
DisGeNet	https://www.disgenet.org/	DisGeNet is a discovery platform containing one of the largest publicly available collections of genes and variants associated with human diseases.
DAVID	https://david.ncifcrf.gov/	DAVID provides a comprehensive set of functional annotation tools for investigators to understand the biological roles of a large list of genes.
IMEx	http://imex.sourceforge.net/	IMEx is a consortium that makes a data resource, which enables the user to download, combine, visualize and analyze data in a single format from multiple resources.
PSI-MI CV	https://www.ebi.ac.uk/ols/ontologies/mi	PSI-MI CV is a structured and controlled vocabulary for the annotation of experiments concerned with PPIs.
VerSeDa	http://genomics.cicbiogune.es/VerSeDa/index.php	VerSeDa has been developed to accelerate the prediction process for whole secretomes (the full set of secreted proteins by a given organism).
UniProtKB	https://www.uniprot.org/	The UniProtKB is the central hub for the collection of functional information on proteins, with accurate, consistent and rich annotation.

DAVID indicates Database for Annotation, Visualization and Integrated Discovery Bioinformatics Resources; IMEX, International Molecular Exchange; PSI, Proteomics Standards Initiative; MI, Molecular Interactions; CV, controlled vocabulary; VerSeDa, Vertebrate Secretome Database; UniProt Knowledgebase, UniProtKB.

Glial secretome refers to the full set of proteins secreted by glial cells. Currently, glial secretome has been a topic of active interest in biomarker discovery (17, 23, 24). Accordingly, a number of proteomics studies have identified numerous secretory proteins related to various neurological disorders (19, 25–29). For instance, amyloid peptides and their precursors, tau proteins (30), have been identified as potential biomarkers for Alzheimer’s disease and alpha-synuclein (31) and apolipoprotein H (32) for Parkinson’s disease (33). Nevertheless, there are many hurdles regarding glial secretome analysis. First, secretome analysis can typically be compromised due to contaminations with proteins from cell debris or culture supplements and result in false identification. Second, it is hard to predict whether a protein is secreted or not, since proteins can be secreted via two pathways, by either the canonical (through an *N*-terminal signal peptide) or non-canonical secretion pathway (34, 35). To overcome these limitations, several bioinformatics tools, such as SignalP (36), SecretomeP (37), TMHMM (38) and WoLF.PSORT (39), have been developed that can predict secretory proteins. However, these tools show varying reliabilities of detection in secretome analysis and can be biased. Therefore, a considerable amount of time and effort should be invested to confirm the results. To our knowledge, no specific database for glial secretome presently exists. Thus, a systematic and curated database that can manage the glial secretome data is in high demand. To fill this important gap, we generated the ‘Gliome’ database, a web-based tool to access and analyze glia-derived secretory proteins. The database provides a set of information about manually curated glia-based experiments as well as the disease associations and protein–protein interactions (PPIs) of glia-secreted proteins. It integrates a diffused glial secretome data and provides a comprehensive platform for glial research.

## Materials and Methods

### Data collection and pre-processing

The data in the Gliome database were manually obtained through a comprehensive literature search at the Google Scholar, Scopus and PubMed databases using general keywords, namely ‘glia, astrocyte, microglia, oligodendrocyte, Schwann cell, secretome and secretory protein’. To collect all the relevant data, literatures were searched by the following selection criteria: (i) main or supplementary tables containing detailed information on glial proteins, which were identified by secretomic analysis such as LC-MS/MS analysis, ELISA or protein array; (ii) differential expression of glia-secreted proteins under specific stimulation conditions; (iii) experiments related to specific secretory proteins; and (iv) molecular/clinical validation experiments have been performed for the identified biomarkers (glia-secreted proteins). These criteria were used to gather information of secretome analysis, expression level under a certain stimulation, experimental design and relevance to clinical use. Applying these criteria, 58 publications were selected as the data source to collect the relevant data (Figure 1). All the glial secretory proteins found through the literature search were compiled for the generation of the Gliome database (Figure 2). For ID mapping, different types of protein IDs (such as the International Protein Index) were unified to the UniProt ID using UniProtKB (40) and the ID conversion tool of the Database for Annotation, Visualization and Integrated Discovery (DAVID) Bioinformatics Resources (41). The basic information [such as signal peptide (a discrimination score obtained using the SignalP. Y indicates discrimination score greater than 0.45; N, discrimination score lesser than 0.45. Secretory proteins are indicated by Y.), SecretomeP value (neural network score obtained using the SecretomeP. Non-classically secreted proteins are predicted based on a score greater than 0.5), functions and Gene Ontology functions] about the glial secretory proteins was annotated using the public databases VerSeDa (42), UniProtKB, PubMed, DisGeNet, Scopus and DAVID (Table 1). For searching the publications regarding the PPIs of the glial secretory proteins, all the synonyms for each protein at UniProtKB and Entrez Gene (43) were used. For PPI detection methods, we used the standard nomenclature in two highly verified databases, International Molecular Exchange (http://imex.sourceforge.net/) and the PSI-MI controlled vocabulary (https://www.ebi.ac.uk/ols/ontologies/mi). Using various combinations of protein names, their synonyms and PPI detection methods, we searched the literature for the physical interactors of glia-secreted proteins. The PubMed and Scopus databases were manually screened for the experimental information related to PPI, whereby 137 articles were identified (Figure 1).

### Database architecture and web interface

The Gliome database used a MySQL database server version 5.7.22 to store and query the collected data. The web interface was implemented by Ruby on Rails version 5.1.4, a server-side web application framework written in Ruby programming language. The system runs on a Phusion Passenger application server version 5.2.3 and an Nginx HTTP server version 1.12.2 hosted on an Ubuntu Linux server version 16.04.2 LTS. HTML5, CSS and JavaScript were used for building the client-side user interfaces. We also utilized jQuery 3.2, Semantic UI version 2.3.1 and Font Awesome version 4.7.0 to generate a user-friendly interface.

## Results

### The structure of the Gliome database

The Gliome database was structured to provide the following: (i) a straightforward searchable depository for glial secretory proteins and basic information about them, such as secretory pathway and functions; (ii) information across different secretome measurements; (iii) disease relevance; and (iv) prediction of the protein interactors of each glial secretory protein. Therefore, the Gliome database comprises glial secretory proteins characterized in various studies and provides information about the annotation of these proteins and predictions about their functions.

**Figure 3:**
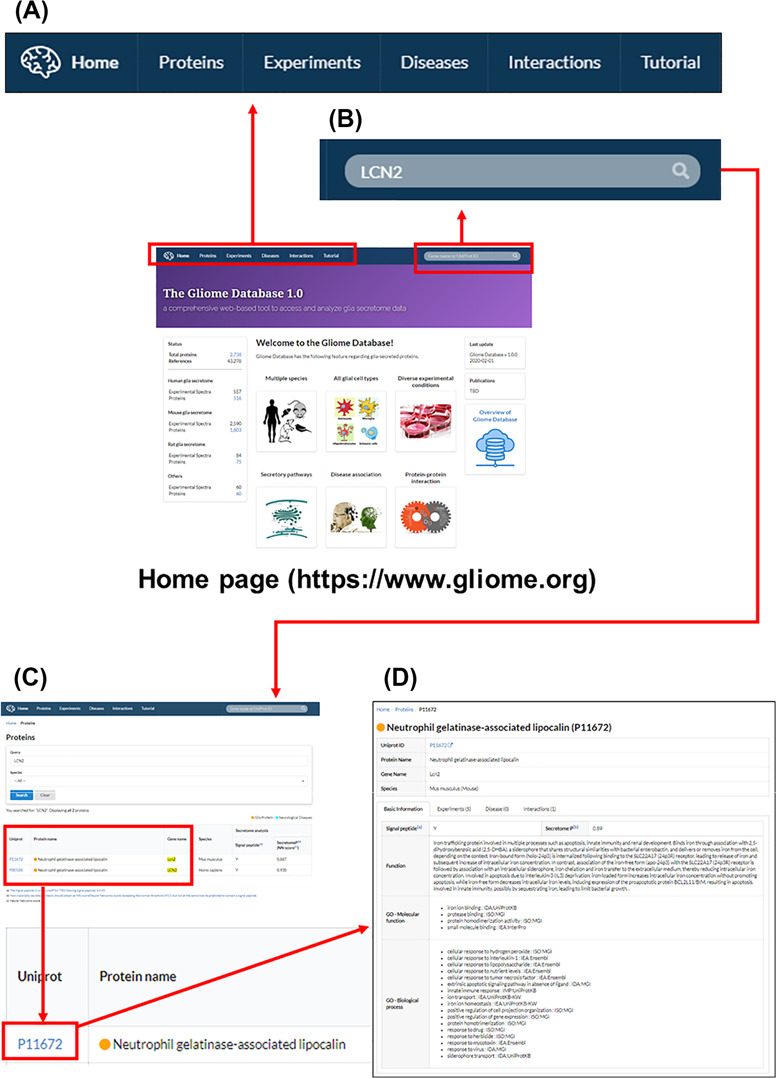
The main page and appearance of the Gliome database. (**A**) Main tabs, (**B**) quick search window, (**C**) protein list, (**D**) protein information. When a user enters a protein name, such as ‘LCN2’, on the quick search window, the page of LCN2 protein appears. By clicking the UniProt ID, the relevant detailed information can be obtained.

**Figure 4:**
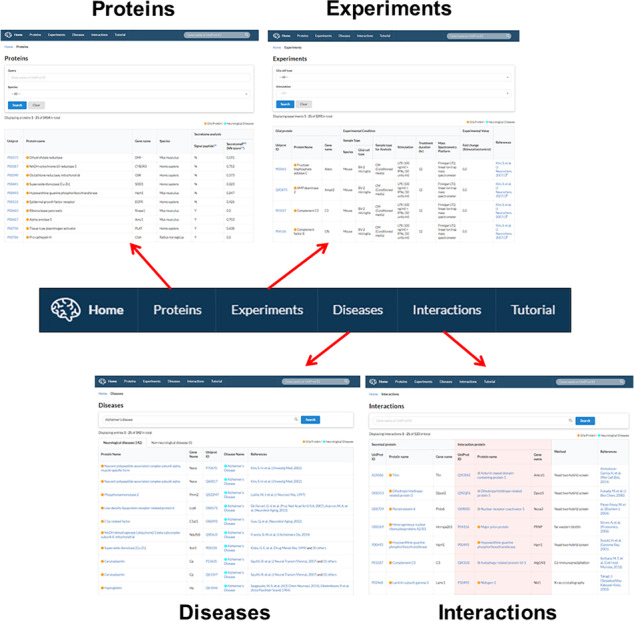
Gliome database navigation. The main tab at the top of the homepage is hyperlinked with four major informative browsers including protein information (‘Proteins’), experiments (‘Experiments’), associated diseases (‘Diseases’) and PPI (‘Interactions’).

### Web interface

The Gliome database home page includes database status and a brief introduction of the database within four major lines: (i) information retrieval about glia-secreted proteins, (ii) analysis of differential protein expression under specific conditions, (iii) disease associations of glia-secreted proteins and (iv) PPIs of glia-secreted proteins (Figures 2 and 3). On top of the home page, we implemented the query window that allows users for a quick protein search (Figure 3). To query, the users first need to specify a gene name or UniProt ID. Entering a search term (gene name or UniProt ID) opens the list table that contains the search term in any field. When users click the UniProt ID in the table, the detailed information about the queried protein appears.

The Gliome database contains four main tabs designed for different purposes. Users can utilize the database by clicking ‘Proteins’, ‘Experiments’, ‘Diseases’ and ‘Interactions’ (Figure 4). The Gliome database also provides instructions in the ‘Tutorial’ section. The browser that appears after clicking ‘Proteins’ tab includes a query box and the whole protein list. Users can choose species using the optional drop-down menu. If users enter a gene name or UniProt ID on the query box and select species using the drop-down menu, a result table will be displayed. By clicking the UniProt ID, users can browse the summary table of basic protein information, including existence (Y)/non-existence (N) of a signal peptide, SecretomeP value, general function and GO functions. At the same time, users can access associated experiments, disease or protein interactions using beside tabs. ‘Experiments’ tab at the top of the main page shows the page of drop-down menus and a complete list of experiments. This page was designed to allow users to browse through the experiments associated with the protein of interest or to simulate an experiment. Users can select a glial cell type and experimental stimulus and browse glial secretory proteins under a selected experimental condition. Clicking the UniProt ID or References returns to the page of protein information or publication at the PubMed database. The ‘Diseases’ tab provides a list of the proteins associated with the queried disease of interest. Entering a disease name (auto-completion) displays the protein names, UniProt IDs and References. The ‘Interactions’ page offers a manually curated protein interaction of each protein and related analytical methods.

## Discussion

The goal of the Gliome database is to answer several important questions. (i) Is the protein of interest secreted? (ii) What is the cellular origin of the secretory protein of interest? (iii) Has the secretory protein of interest been experimentally investigated? (iv) Is the protein of interest relevant to human diseases? (v) Is there any protein that interacts with the secretory protein of interest?

Our database provides well-summarized fundamental information on glia-secreted proteins. The basic information may be useful to predict the biological functions of novel glial proteins and to determine whether a protein of interest has a canonical or non-canonical secretory pathway. Secretory proteins were grouped according to glial cell types and stimulation conditions in the ‘Experiments’ tab so that one can easily identify the glial cell type and experimental condition from which a particular protein of interest was derived. Moreover, as the glial secretome reflects various states of the nervous system under a given condition in real time (24), it is a potentially rich source of biomarkers. Accordingly, a set of glial secretory proteins associated with diseases may be a fundamental source for disease-specific biomarkers or therapeutic targets (44). Recently, the role of glia-derived exosome or extracellular vesicle (EV) proteins in neuron–glia communication and neurological disease has been emerging (45, 46). EVs from ALS patients and animal models contained many misfolded proteins mainly released by astrocytes, implying the deleterious role of astrocytes in ALS pathology (47), and the stimulation of astrocytes with proinflammatory or anti-inflammatory cytokines results in the release of neurotoxic or neuroprotective proteins in EVs, respectively (48). Therefore, our database provides information about a broad range of glial secretome.

Furthermore, our database provides information about the PPIs of glia-secreted proteins. PPI plays a fundamental role in virtually all biological processes. The identification of the interacting proteins of specific glia-secreted proteins may deepen our understanding of the mechanisms by which the glial proteins act and glia regulate the nervous system. This will lead to the discovery of mechanism-based drug targets and the optimization of treatment strategies (49). In our database, the manually curated PPI information was included together with other public PPI databases, such as IntAt, MINT and STRING, to provide high-confident experimental information. Therefore, the Gliome database is equipped with more accurate PPI information of glia-secreted proteins, which may be helpful for clinical usage in the future.

## Funding

This research was supported by Basic Science Research Program through the National Research Foundation, funded by the Korean government (Ministry of Science, ICT and Future Planning, MSIP) (2016M3C7A1904148). DK was supported by the Collaborative Genome Program for Fostering New Post-Genome Industry of the National Research Foundation (NRF) funded by the Ministry of Science and ICT (MSIT) (NRF-2014M3C9A3064706). 

*Conflict of interest*. The authors declare no competing interest.

**Database URL:** www.gliome.org
